# Fitness, physical activity, and exercise in multiple sclerosis: a systematic review on current evidence for interactions with disease activity and progression

**DOI:** 10.1007/s00415-021-10935-6

**Published:** 2022-01-27

**Authors:** Sebastian Proschinger, Puya Kuhwand, Annette Rademacher, David Walzik, Clemens Warnke, Philipp Zimmer, Niklas Joisten

**Affiliations:** 1 Department for Molecular and Cellular Sports Medicine, Institute of Cardiovascular Research and Sports Medicine, German Sport University Cologne, Cologne, Germany; 2 Medical Faculty, University Hospital Cologne, Cologne, Germany; 3 Marianne-Strauß-Klinik, Behandlungszentrum Kempfenhausen Für Multiple Sklerose Kranke gGmbH, Berg, Germany; 4 Division of Performance and Health (Sports Medicine), Institute for Sport and Sport Science, TU Dortmund University, Dortmund, Germany; 5 Department of Neurology, University Hospital Cologne, Cologne, Germany

**Keywords:** Physical exercise, Evidence based, Neurorehabilitation, Physical activity, Magnetic resonance imaging, Systematic review

## Abstract

**Background:**

A moderate to high level of physical activity, including regular exercise, represents an established behavioral and rehabilitative approach for persons with multiple sclerosis (pwMS). Although being increasingly proposed to limit disease activity and progression, high-quality evidence is lacking.

**Objective:**

The objective of the study is to provide valuable information for MS clinicians and researchers by systematically evaluating the current state of evidence (i) whether exercise interventions affect established clinical measures of disease activity and progression in pwMS (i.e., EDSS, relapse rate, lesion load, brain volume, MSFC) and (ii) how the physical activity and fitness level interact with these measures.

**Methods:**

Literature search was conducted in MEDLINE, EMBASE, CINAHL, and SPORTDiscus. Evaluation of evidence quality was done based on standards published by The American Academy of Neurology.

**Results:**

It is likely that exercise improves the MSFC score, whereas the EDSS score, lesion load, and brain volume are likely to remain unchanged over the intervention period. It is possible that exercise decreases the relapse rate. Results from cross-sectional studies indicate beneficial effects of a high physical activity or fitness level on clinical measures which, however, is not corroborated by high evidence quality.

**Conclusions:**

A (supportive) disease-modifying effect of exercise in pwMS cannot be concluded. The rather low evidence quality of existing RCTs underlines the need to conduct more well-designed studies assessing different measures of disease activity or progression as primary end points. A major limitation is the short intervention duration of existing studies which limits meaningful exercise-induced effects on most disability measures. Findings from cross-sectional studies are difficult to contextualize regarding clinical importance due to their solely associative character and low evidence quality.

**PROSPERO registration number:**

CRD42020188774.

**Supplementary Information:**

The online version contains supplementary material available at 10.1007/s00415-021-10935-6.

## Introduction

Multiple sclerosis (MS) is an early-onset immune-mediated neuroinflammatory disease that leads to progressive neurodegeneration and a wide spectrum of disorders in functional systems [1]. The prevalence has increased substantially in many regions since 1990, reaching approximately 2.8 million persons with MS (pwMS) worldwide in 2020 [2]. Most disease-modifying therapies use medication strategies that downregulate immune activation to halt disease progression, prevent relapses, or to partly reverse disability [3]. However, these therapies comprise side effects such as an increased risk of secondary immunosuppression, thereby increasing the likelihood to acquire mild to serious infections. Therapies have been registered in monotherapy only, while other non-pharmaceutical interventions without known side effects might have added benefits.

For decades, physical exercise was not recommended by neurologists and leading MS institutions according to the general assumption that exercise increases the risk of exacerbations and symptoms of fatigue. Research over the past 25 years, however, revealed that well-structured exercise programs are feasible, safe and a useful (supportive) treatment strategy to alleviate symptoms in pwMS [4]. Therefore, physical exercise gained extensive interest in MS rehabilitation [5, 6]. Peripheral biomarkers such as the matrix metalloproteinase-2, a well-known marker for blood-barrier breakdown in neuroinflammatory diseases including MS [7], can be reduced after 3 weeks of high-intensity exercise [8], while other studies revealed an increase in serum levels of the brain-derived neurotrophic factor after different exercise regimens [9]. Of high clinical relevance are imaging-based measures of disease activity and progression such as changes in T2 hyperintense and gadolinium-enhancing T1 lesion load or brain volume, respectively [10]. During the recent years, these measures have been increasingly assessed in clinical exercise studies [11, 12]. Indeed, it was shown that gray and white matter volumes were increased in an aged population after 6 months of aerobic exercise [13]. Other established measures are the Expanded Disability Status Scale (EDSS), the annualized relapse rate, and the Multiple Sclerosis Functional Composite (MSFC) [10]. Preclinical evidence from animal models of experimental MS supports the beneficial exercise-induced effects on disease-specific clinical measures such as the myelination status, axonal integrity, disease onset and disease progression [14–16].

Evidence-based guidelines have been developed to increase the level of physical activity in pwMS [17]. However, a recent meta-analysis showed that this population is still physically less active than the healthy population [18]. The importance of an active lifestyle on health benefits in healthy and diseased populations has been extensively reviewed [19, 20] and is underscored by results from cross-sectional studies that report negative associations between higher physical activity or fitness levels and clinical measures of disease activity or progression in pwMS [21–23]. The concept of physical activity can be defined as any bodily movement initiated by skeletal muscle contraction that leads to energy expenditure and includes the two domains *lifestyle physical activity* (planned or unplanned leisure, occupational, or household activities) and *exercise* (performed repeatedly over an extended period of time with a specific external objective) [24]. From here on, the term *exercise* refers to bodily movements within a structured exercise intervention of a study, whereas the term *physical activit*y considers both domains which are assessed by actigraphy or questionnaire. The term *fitness* is used throughout the article to refer to the cardiorespiratory fitness (CRF) (i.e., VO_2peak_ and VO_2peak_) or strength outcomes (i.e., maximum strength measures) [25]. A short summary of the respective terms can be found in Table 1.

**Table 1:** Definitions of the terms physical activity, fitness, and exercise [24, 25]

Exercise
A form of *physical activity* that is usually performed repeatedly over an extended time period and in a structured way to reach specific objectives such as health or performance improvements
Physical activity
Any bodily movement initiated by skeletal muscle contraction that leads to energy expenditure that is reached by either *exercise* or *lifestyle physical activity* (daily accumulation of at least 30 min of leisure, occupational, or household activities being at least moderate to vigorous in their intensity)
Fitness
A set of attributes (i.e., cardiorespiratory fitness, muscular strength) relating to the ability to perform *physical activity*

Despite the good evidence of exercise-induced disease-modifying effects observed in preclinical animal models and reviews that address the medical role of exercise in MS [6, 26], focusing on aspects of tertiary, secondary, and primary prevention, this review aims to systematically summarize the current state and quality of evidence, based on standards published for therapeutic trials by The American Academy of Neurology (AAN), on whether (i) exercise interventions affect established clinical measures of disease activity and progression (i.e., EDSS, relapse rate, lesion load, brain volume, and MSFC) in pwMS and (ii) how the physical activity and fitness level interact with these measures. The results will be discussed in the context of methodological and conceptual limitations, providing valuable information for MS clinicians as well as for researchers in this field.

## Methods

This study was conducted in accordance with the Preferred Reporting Items for Systematic reviews and Meta-Analyses (PRISMA) [27]. The protocol was pre-registered on PROSPERO (registration number: CRD42020188774).

### Search strategy

The databases MEDLINE (via PubMed), EMBASE, CINAHL, and SPORTDiscus were used for electronic literature search from inception until January 31, 2021. The search strategy included Medical Subject Headings (MeSH) and text words of the defined MS population, surrogate terms of exercise, physical activity, and fitness, as well as clinical measures of disease activity and progression. The categories were combined through Boolean operators (‘‘AND’’, ‘‘OR’’) (Table S1 for complete search string). Titles and abstracts were screened by two independent reviewers (S.P., P.K.). Only peer-reviewed articles published in English language were included. Covidence Review Software, recommended by the Cochrane Collaboration, was used for the review process.

### Selection criteria

#### Population

Adult pwMS (≥ 18 years) were included regardless of the stage or clinical subtype of disease.

#### Intervention and comparison

Intervention studies that conducted endurance, resistance, balance, or mind–body exercise with (usual care/passive) and without controls were included. Interventions that are of a predominantly supportive character (i.e., functional electrical stimulation cycling, robot-assisted gait exercises) or mainly consider task-oriented concise limb movements to improve activities of daily living (i.e., grasping, pinching) were not considered.

Further, non-interventional cross-sectional studies assessing the physical activity (by actigraphy or questionnaire) and/or fitness level (i.e., CRF, strength) were included.

#### Outcomes

Studies reporting at least one clinical measure of disease activity or progression, i.e., EDSS score, relapse rate, lesion load, brain volume, or the MSFC score, were included. The EDSS is commonly used by neurologists and, according to an ordinal scale ranging from 0 (normal neurological examination) to 10 (death due to MS), describes symptoms and signs in eight functional systems [10]. The MSFC was developed by the MS Society’s Clinical Assessment Task Force as an additional clinical measure of disability progression and comprises two motor and one cognitive test [28]. Results correlate with several clinically relevant measures such as EDSS change, relapse rate, white matter lesion load, and various atrophy measures [10].

Studies investigating solely potential underlying cellular or molecular mechanisms, e.g., changes in immune cell subsets, cytokines, and neurotrophic factors, were not considered.

#### Study design

**Fig. 1:**
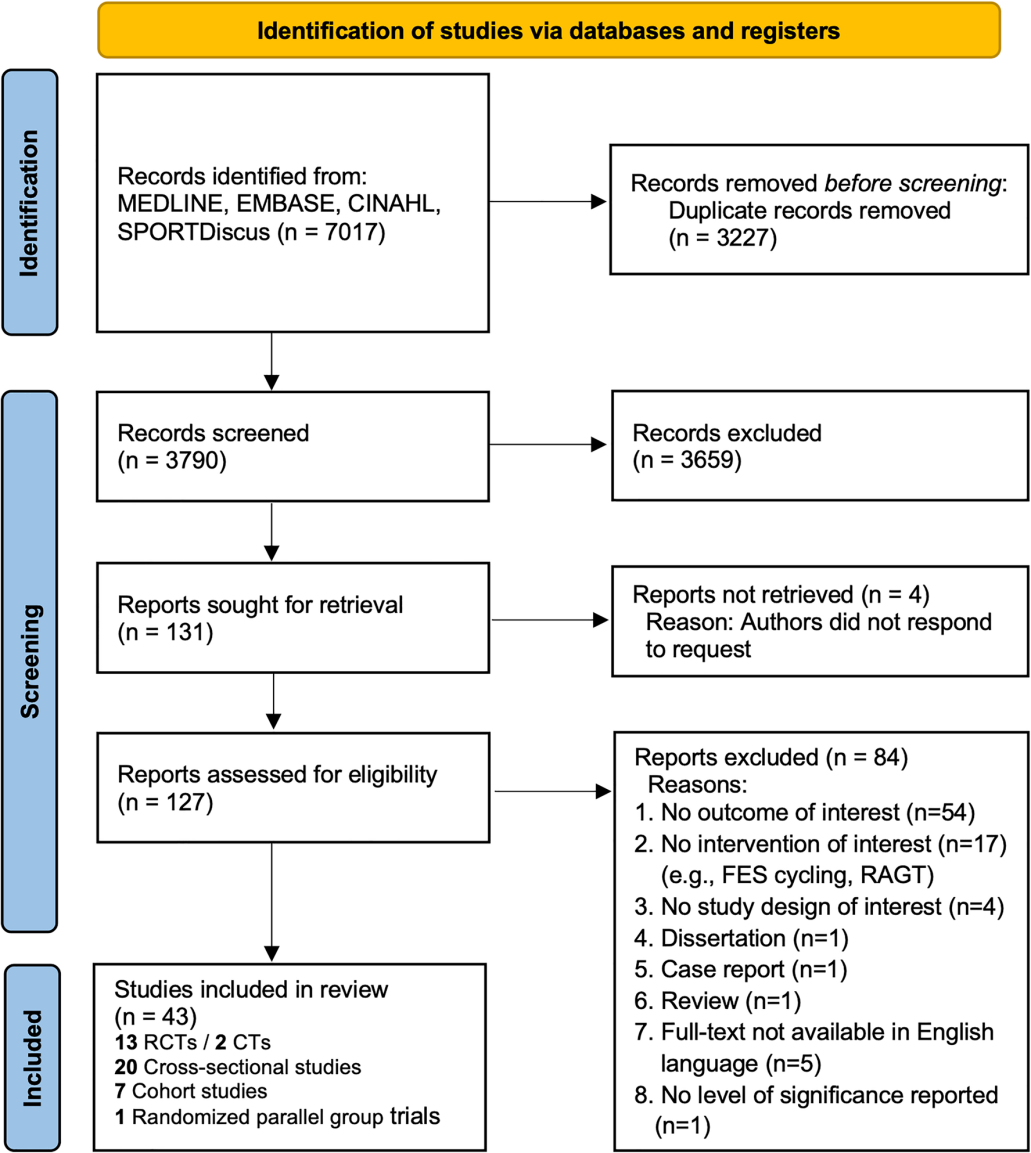
PRISMA flow diagram of literature search and results. FES: functional electrical stimulation cycling, RAGT: robot-assisted gait exercises, RCT: randomized controlled trial, CT: controlled trial

Longitudinal (randomized) controlled trials ((R)CTs), non-controlled cohort studies and cross-sectional studies were included. All other types of articles (e.g., case reports, reviews, opinion articles) were excluded. The process of study selection is shown in Fig. 1.

### Quality assessment, rating of evidence and development of recommendations

Two independent reviewers (S.P., P.K.) screened the studies for eligibility and methodological quality. The Cochrane Risk of Bias (RoB) tool was used to assess the risk of various bias domains (i.e., selection, reporting, performance, detection, and attrition) for included RCTs (Table S2) [29]. Each domain was judged as ‘high RoB’, ‘unclear RoB’, or ‘low RoB’. Decisions on the studies´ evidence level classification (classification I to IV) were based on standards published for therapeutic trials by the AAN (Table 2) [30]. According to the strength of evidence for respective outcomes in each research area (i.e., exercise, physical activity, fitness) based on the level of confidence in evidence (*high confidence, moderate confidence, low confidence, very low confidence*) [30], evidence-based recommendations will be provided, classified as *highly likely to be effective, ineffective or harmful* (level A), *likely to be effective, ineffective or harmful* (level B), *possibly likely to be effective, ineffective or harmful* (level C), or *data is insufficient or too conflicting to conclude an effect* (level U) (Table 2).

**Table 2:** The American Academy of Neurology´s Classification of Evidence (I, II, III, IV) and level of confidence/classification of recommendations (A, B, C, U)

A	Classification of evidence	
	I	Triple-masked RCT in a representative population. Relevant baseline characteristics are presented, substantially equivalent or there is appropriate statistical adjustment. Additional criteria: A) concealed allocation, B) no more than two primary outcomes specified, C) exclusion/inclusion criteria clearly defined, D) at least 80% of participants completing the study
	II	RCT that lacks one or two Class I criteria A–D or prospective matched cohort study with masked/objective outcome assessment that meets B–D. Randomized crossover trials reporting either period and carryover effects or baseline characteristics of treatment order groups. Relevant baseline characteristics are presented, substantially equivalent or there is appropriate statistical adjustment
	III	Controlled studies (including studies with external controls) or crossover trial missing both period/carryover effects and presentation of baseline characteristics. Outcome is independently assessed, or independently derived by objective outcome measurement
	IV	Studies not meeting Class I, II, or III criteria
B	Level of confidence/classification of recommendations	
	A	High confidence: highly likely to be effective, ineffective or harmful (or [not] useful/predictive) for the given condition in the specified population. Requires at least two Class I studies
	B	Moderate confidence: likely to be effective, ineffective or harmful (or [not] useful/predictive) for the given condition in the specified population. Requires one Class I study or at least two Class II studies
	C	Low confidence: possibly likely to be effective, ineffective or harmful (or [not] useful/predictive) for the given condition in the specified population. Requires one Class II study or at least two Class III studies
	U	Very low confidence: data insufficient or conflicting, given current knowledge, treatment is unproven

In cases of disagreement considering eligibility, methodological quality, or classification of evidence level, conflicts were resolved by consensus. If no consensus could be achieved, a third reviewer (N.J.) was consulted who was blinded to the judgments of the first two reviewers. Inter-rater correlation coefficients are 89.4% and 94.4% for AAN and Cochrane RoB, respectively.

All relevant study characteristics are synthesized and presented in Tables 3, 4 and 5.

**Table 3:** Study characteristics of included randomized and non-randomized controlled trials

**Author, year**	**Study population ** N (f/m), MS phenotype (R/S/P), Age (y), EDSS, DD (y)		**AAN Class**	**MS-specific outcome ** (primary/secondary)	**Intervention**	**Interaction effects**	**Risk of bias**^d^
			**Randomized controlled trials**				
Bjarnadottir, 2007	IG	CG (passive)	III	EDSS (NR)	5 weeks 3x/week 60 min session progressive cycling (15-20 min) at ~ 55% VO2_peak_ and progressive resistance exercise (13 exercises)	n.s	2–2–2
	6 (3/3), R, 38.7 y, EDSS 2.1, DD 8.7 y	10 (8/2), R, 36.1 y, EDSS 1.8, DD 8.3 y					
De Oliveira, 2016	IG	CG (waitlist)	II	EDSS (NR)	24 weeks 1x/week 60 min Yoga	n.s	3–3–0
	6 (6/0), NR, 46 y (8), EDSS 3.2 (1.2), DD NR	6 (5/1), NR, 45 y (9), EDSS 3.1 (1.9), DD NR					
Feys, 2019	IG	CG (waitlist)	III	Total and region-specific brain volume (secondary)	12 weeks 3x/week (min NR) progressive running	↑ left pallidum volume	1–4–1
	21 (20/1), NR, 36.6 y (8.5), EDSS NR, DD 8.1 y (6.1)	21 (18/3), NR, 44.4 y (8.5), EDSS NR, DD 9.2 y (5.3)					
Golzari, 2010	IG	CG (passive)	III	EDSS (NR)	8 weeks (24 sessions, min NR) progressive combined stretching, aerobic and resistance exercises	No interaction effects assessed	0–5–1
	10 (10/0), R, 32.15 y (7.57), EDSS 2.14 (1.06), DD NR	10 (10/0), R, 33.75 y (8.18), EDSS 1.95 (1.06), DD NR					
Grazioli, 2019	IG	CG (usual care)	III	EDSS (secondary)	24 weeks 2x/week 60 min combined cycling at 65% HR_max_ and strength training at 50% 1RM	n.s	4–2–0
	10 (7/3), NR, 45.91 y (12.09), EDSS 4.73 (.9), DD NR	10 (8/2), NR, 39.40 y (10.26), EDSS 4.40 (2.26), DD NR					
Hoang, 2016	IG	CG (passive)	II	MSFC (secondary)	12 weeks 2x/week 30 min interactive exergaming (step training)	↑ MSFC	5–0–1
	28 (21/7), R/S/P, 53.4 y (10.7), EDSS 4.1 (1.4), DD NR	22 (17/5), R/S/P, 51.4 y (12.8), EDSS 4.2 (1.2), DD NR					
Kjølhede, 2018^e^	IG	CG (Waitlist)	II	Total and region-specific brain volume, lesion load, cortical thickness, PBVC, MSFC (NR)	24 weeks 2x/week (min NR) progressive machine-based resistance training	↑ relative cortical thickness, MSFC	5–0–1
	18 (NR)	17 (NR)					
	R, 43 y (8), EDSS 2.9 (0.2), DD 7 y (7)						
Langeskov-Christensen, 2021	IG	CG (waitlist)	II	PBVC (primary), volumes of various brain regions, upper spinal cord volume cortical thickness, T2 and black hole lesion load, relapse rate	24 weeks 2x/week 30–60 min progressive aerobic exercise at 65–95% HR_max_	↓ annualized relapses	5–0–1
	43 (26/17), R/S/P, 44 y (9.5), EDSS 2.7 (1.4), DD 10.9 y (7.9)	43 (26/17), R/S/P, 45.6 y (9.3), EDSS 2.8 (1.6), DD 8.6 y (6)					
Miller, 2011	IG	CG (usual care)	II	EDSS (secondary)	8 weeks 2x/week 60 min task-specific physiotherapy (strength exercises for upper and lower body)	n.s	5–1–0
	15 (11/4), S/P, 56.3 y (9.0), EDSS 7 (0.5), DD 13 y (9.1)	15 (8/7), S/P, 52.9 y (6.3), EDSS 7.1 (0.8), DD 18.7 y (8.1)					
Moradi, 2015	IG	CG (passive)	III	EDSS (NR)	8 weeks 3x/week (min NR) progressive machine-based resistance training at 50–80% 1RM	↓ EDSS	5–1–0
	8 (0/8), R/S, 34.38 (11.07) y, EDSS 3 (1–6)^a^, DD 8.12 y (4.79)	10 (0/10), R/S, 33.13 (7.08) y, EDSS 3 (1–5)^a^, DD 6.5 y (5.78)					
Petajan, 1996	IG	CG (waitlist)	III	EDSS (NR)	15 weeks 3x/week 40 min progressive combined arm/leg ergometry at 60% VO_2max_	n.s	3–3–0
	21 (15/6), NR, 41.1 y (2), EDSS 3.8 (0.3), DD 9.3 y (1.6)	25 (16/9), NR, 39 y (1.7), EDSS 2.9 (0.3), DD 6.2 y (1.1)					
Romberg, 2005	IG	CG (waitlist)	III	MSFC, EDSS (NR)	26 weeks 3-4x/week (min NR) progressive aerobic (1x/week) and resistance exercise (2-3x/week)	↑ MSFC	2–2–2
	47 (30/17), NR, 43.8 y (6.3), EDSS 2 (1.5–3.5)^b^, DD 6 y (6.5)	48 (31/17), NR, 43.9 y (7.1), EDSS 2.5 (2–3.5)^b^, DD 5.5 y (6.4)					
Sangelaji, 2014	IG	CG (waitlist)	III	EDSS (NR)	10 weeks 3x/week 60–90 min progressive combined cycling, running, resistance and balance exercises	n.s	2–3–1
	39 (24/15), NR, 33.05 y (7.68), EDSS NR, DD NR	22 (15/7), NR, 32.05 y (6.35), EDSS NR, DD NR					
			**Controlled trials**				
Niwald, 2017	IG	CG (NR)	III	EDSS (NR)	4 weeks 5x/week (3 × 10 min/day) progressive cycling	n.s	n.a
	21 (13/8), NR, 57.19 y (7.62), EDSS 5–6.5^c^, DD 13.9 y (11.45)	32 (21/11), NR, 59.7 y (4.2), EDSS 5–6.5^c^, DD 12.2 y (2.6)					
Rasova, 2006	Group 1: 24, NR, NR, EDSS 4.10, DD NR Group 2: 36, NR, NR, EDSS 2.21, DD NR Group 3: 19, NR, NR, EDSS 3.42, DD NR	Group 4 (passive) 16, NR, NR, EDSS 2.32, DD NR	III	EDSS (NR)	Group 1: 8 weeks 2x/week 60 min neurophysiologically based physiotherapy Group 2: 8 weeks 2x/week 2-30 min progressive cycling at ~ 60% VO_2max_ Group 3: combined program of group 1 and 2	↓ EDSS (Group 1, 2, and 3 vs. 4)	n.a

Data presented as mean (SD) unless otherwise noted. ↑: increased, ↓: decreased

*AAN* American Academy of Neurology, *CG* control group, *DD* disease duration, *EDSS* Expanded Disability Status Scale, *f* female, *HR*_*max*_ maximum heart rate, *IG* intervention group, *m* male, *MS* multiple sclerosis, *MSFC* multiple sclerosis functional composite, *n.a.* not applicable, *n.s.* not significant, *NR* not reported, *P* primary progressive MS, *PBVC* percentage brain volume change, *R* relapsing–remitting MS, *RM* repetition maximum, *RSFC* resting-state functional connectivity, *S* secondary progressive MS, *VO*_*2peak/max*_ peak/maximum oxygen consumption, *y* years

^a^Median (range)

^b^Median (IQR)

^c^Range

^d^Left score: “low risk of bias”; middle score: “unclear”; right score: “high risk of bias”

^e^Referred to as crossover design by the study authors. Due to design issues, results are only partially considered here

**Table 4:** Study characteristics of included cross-sectional studies

**Author, year**	**Study population ** N (f/m), MS phenotype (R/S/P/prog.), Age (y), EDSS, DD (y)	**AAN Class**	**MS-specific outcome ** (primary/secondary)	**Fitness/activity outcome** (assessment)	**Results**
Block, 2017	99 (63/36), R/S/P, 50.1 y (13.8), EDSS 4.1 (0–6.5)^d^, DD 13 y (6–21)^b^	IV	EDSS (NR)	PA level (Fitbit^®^)	EDSS associated with average daily step count (*r* = − 0.71)
Cavanaugh, 2011	21 (12/9), NR, 57.6 y (12.7), EDSS 3.5—7.5^c^, EDSS ≤ 4.5: DD 11 y (12)/EDSS ≥ 5: DD 17.3 y (7.1)	IV	EDSS (NR)	PA level (step activity monitor)	EDSS associated with total daily step count (*r* = −0.90)
Chaves, 2019	82 (58/24), R/S/P, 47.40 y (10.2), EDSS 2.04 (1.7), DD 13.10 y (8.0)	IV	EDSS (NR)	Aerobic capacity (CPET on a TBRS)	Cortical silent period (intracortical inhibition) associated with VO_2max_ (*r* = − 0.41)
Fjeldstad, 2015	13 (9/4), R, 47.6 y (3.0), EDSS 2.5 (0.5), DD 7.5 y (1.0)	III	EDSS (primary)	PA level (ActiGraph^®^)	EDSS associated with weekly step count (*r* = − 0.68) and minutes spent in PA (*r* = − 0.61)
Foglio, 1994	24 (17/7), NR, 48 y (9), EDSS 5.3 (2), DD 12.2 y (6)	IV	EDSS (NR)	Aerobic capacity (CPET on an arm ergometer)	n.s
Fritz, 2017	29 (17/12), R, 48.7 y (11.5), EDSS 4.0 (1.0–6.5)^a^, DD 11.9 y (8.7)	III	EDSS, Spinal cord area (NR)	Maximal strength (summation of hip flexor/extensor/abductor strength with dynamometer)	Strength score associated with corticospinal tract FA (*r* = 0.26), spinal cord area (*r* = 0.33), magnetization transfer ratio (*r* = 0.29)
Heine, 2015	116 (70%/30%), R/S/P, 44.4 y (9.7), EDSS 2.7 (1.3), DD NR	IV	EDSS (NR)	Aerobic capacity (CPET)	EDSS associated with VO_2peak_ (*r* = − 0.418)
Kalron, 2019	289 (176/113), R/prog., 41.2 y (12.9, EDSS 2.0 (0–6.5)^a^, DD 6.8 y (8.4)	IV	EDSS (NR)	PA level (GLTEQ)	Sig. difference in GLTEQ score between i) EDSS ≤ 1.5 and EDSS 4–5.5 + EDSS ≥ 6; ii) EDSS 2–3.5 and EDSS 4–5.5 + EDSS ≥ 6
Kalron, 2020	153 (104/49), R/S/P, 39.3 y (12.0), EDSS 2.0 (0—6.5)^a^, DD 6.6 y (8.9)	IV	EDSS, volume of subcortical brain regions (NR)	PA level (GLTEQ)	Sig. difference in EDSS and volume of right hippocampus between active and inactive group
Kerling, 2014	60 (44/16), NR, 44.0 y (10.4), EDSS ≤ 3 n = 38/EDSS 3.5—6 n = 22, DD NR	III	EDSS, MSFC (NR)	Aerobic capacity (CPET on bicycle ergometer), maximum strength (knee flexor/extensor) isokinetics	Sig. difference in VO_2peak_ and maximal strength between EDSS ≤ 3 vs. EDSS 3.5–6
Klaren, 2015	39 (30/9), R/S/P, 48.7 y (9.6), EDSS 4.5 (2.5)^b^, DD 10.3 y (8.5)	IV	Region-specific brain volume (NR)	PA level (ActiGraph^®^)	MVPA associated with volumes of normalized white & gray matter, thalamus, caudatus, putamen, hippocampus, and pallidum (*r* = 0.37–0.54)
Konečný, 2007	35 (28/7), R/S/P, 49.1 y (10), EDSS 3.0 (1.2), DD 15.4 y (12.5)	IV	EDSS (NR)	Aerobic capacity (CPET on a bicycle ergometer)	EDSS associated with VO_2peak_ (*r* = − 0.47)
Madsen, 2019	242 (147/95), R/S/P, 46.9 y (11.4), EDSS 4.4 (1.3), DD 10.7 y (8.6)	IV	EDSS (NR)	Aerobic capacity (CPET on a bicycle ergometer)	EDSS associated with VO_2peak_ (*r* = − 0.465)
Merkelbach, 2011	80 (57/23), R/S, 43.2 y (9.8), EDSS 3.1 (1.6), DD 8.0 y (6.8)	IV	EDSS (NR)	PA level (Actiwatch^®^)	EDSS associated with mean 24 h PA (*r* = − .471)
Motl, 2015	35 (71%/29%), R/S/P, 50.8 y (9.8), EDSS 5.0 (3.5)^b^, DD 11.4 y (7.5)	IV	Volumes of various deep gray matter structures (NR)	Aerobic capacity (CPET on a recumbent seated stepper)	VO_2peak_ associated with volumes of caudate (*r* = 0.47), putamen (*r* = 0.44), pallidum (*r* = 0.40), hippocampus (*r* = 0.42)
Pilutti, 2015	64 (71.9%/28.1%), R, 52.0 y (7.8), EDSS 4.25 (2.5), DD 13.2 y (8.8)	IV	EDSS (NR)	Aerobic capacity (CPET on an arm ergometer & recumbent stepper), maximal strength (Peak torque of knee extensors & flexors with dynamometer)	EDSS associated with VO_2peak_ (ηρ^2^ = 0.32), knee extensor strength (ηρ^2^ = 0.39) and knee flexor strength (ηρ^2^ = 0.38)
Prakash, 2010	21 (21/0), R, 44.2 y (1.9), EDSS 2.2 (0–6)^d^, DD 7.3 y (0.1)	III	Lesion load volume, brain gray matter atrophy (NR)	Aerobic capacity (CPET on a bicycle ergometer)	VO_2peak_ associated with lesion load volume (*r* = − 0.44), and gray matter volume/white matter FA in various brain regions
Rasova, 2005	112 (83/29), R/S/P, 36.44 y (9.52), EDSS 3.07 (1.68), DD 8.79 y (6.46)	IV	EDSS (NR)	Aerobic capacity (CPET on a bicycle ergometer)	EDSS associated with various cardiorespiratory fitness outcomes (i.e. relative VO_2_ *r* = − 0.46)
Romberg, 2004	96 (61/34), R/S/P, ♀: 43.5 y (6.6), ♂: 44.4 y (6.8), EDSS ♀: 2.2 (0.9), ♂:3.0 (1.2), DD ♀: 5.8 y (6.6), ♂: 5.7 y (6.2)	IV	EDSS (NR)	Aerobic capacity (CPET on a bicycle ergometer)	EDSS associated with relative VO_2peak_ (♀: *r* = − 0.25; ♂: *r* = − 0.50)
Shema-Shiratzky, 2020	44 (♀: 73%), R, 49.2 y (10.7), EDSS 3.5 (2.5–5.0)^b^, DD 13.3 y (9.3)	III	EDSS (NR)	PA level (Axivity AX3^®^)	EDSS associated with step count (*r* = − 0.530) and total daily activity (*r* = −0.337)

Data presented as mean (SD) unless otherwise noted

*χ*^*2*^ chi squared, *ηρ*^*2*^ partial eta squared, *AAN* American Academy of Neurology, *CPET* cardiopulmonary exercise testing, *DD* disease duration, *EDSS* Expanded Disability Status Scale, *f* Female, *FA* fractional anisotropy, *GLTEQ* Godin leisure-time exercise questionnaire, *m* male,* MS* multiple sclerosis, *MSFC* multiple sclerosis functional composite, *MVPA* moderate to vigorous physical activity, *n.s.* not significant, *NR* not reported, *P* primary progressive MS, *PA* physical activity, *R* relapsing–remitting MS, *r* correlation coefficient, *S* secondary progressive MS, *TBRS* total body recumbent stepper, *VO*_*2peak/max*_ peak/Maximum oxygen consumption, *y* years

^a^Median (range)

^b^Median (IQR)

^c^Range

^d^Mean (range)

**Table 5:** Study characteristics of included cohort studies and randomized parallel group trials

**Author, year**	**Study population** N (f/m), MS phenotype (R/S/P), Age (y), EDSS, DD (y)		**AAN Class**	**MS-specific outcome** (primary/secondary)	**Fitness/activity outcome ** (assessment)	**Intervention**	**Results**
			**Cohort studies**				
Bahmani, 2018	18 (15/3), R, 34.29 y (3.21), EDSS 2.05 (1.78), DD newly diagnosed		IV	EDSS (NR)	PA level (IPAQ, short version)	Two-year follow-up activity measurement	EDSS associated with walking time as sub-domain (*r* = − 0.63)
Block, 2019	79 (30/49), R/P, 50.3 y (13.7), 4.0, EDSS (2.5–6.0)^a^, DD 13 y (5.5–20.5)^a^		IV	EDSS (NR)	Daily step count (Fitbit^®^)	One-year follow-up activity measurement	EDSS associated with average daily step count (*β* = − 22.35)
Ertekin, 2012	31 (16/15), R/S/P, 43.6 y (8.2), EDSS 4.62 (1.29), DD 1–10 y *n* = 21/ > 10 y *n* = 10		IV	EDSS (NR)	Not assessed	12 weeks 5x/week 20-25 min progressive strength and balance exercise	n.s.
Ertekin, 2013	17 (13/4), R/S/P, 45.2 y (8.6), EDSS 4.9 (1.7), DD 1–10 y *n* = 7/ > 10 *n* = 10		IV	EDSS (NR)	Not assessed	12 weeks 3/week 35-40 min progressive strength, balance, coordination & functional exercises	n.s
Konečný, 2010	15 (12/3), R/S/P, 50.7 y (13.1), EDSS 2.8 (0.7), DD 15.7 y (14.4)		IV	EDSS (NR)	Aerobic capacity (CPET on a bicycle ergometer), strength (1RM)	8 weeks 2x/week combined cycling (20-40 min week 1–2, 25 min week 3–8) and resistance training (15-20 min)	n.s
Shammas, 2014	11 (7/4), R, S, P, 41 y (9.3), EDSS 3.6 (1.66), DD 12.18 y (10.67)		IV	EDSS (NR)	PA level (MOVE II^®^ triaxial accelerometer)	Activity measurement (one-year period), 10 days every three months	EDSS associated with total step count (*r* = − 0.54)
Stuart, 2020	56 (30/26), S, P, 53.8 y (8.0), EDSS 5.7 (1.3), DD 12.2 y (8.6)		IV	Brain volume change, EDSS (NR)	PA level composite score (SenseWear^®^Armband)	Activity measurement (2.5-year period), 6 days every six months	Annual percentage brain volume change associated with annual change in PA composite score (*r* = 0.357)
			**Randomized parallel group trials**				
Velikonja, 2010	Sports climbing	Yoga	IV	EDSS pyramidal function score (NR)	Not assessed	10 weeks 1x/week (min NR) sports climbing or Yoga	↓ EDSS pyramidal function score within the Sports climbing group
	10, R/S/P, 42 y, EDSS 4, DD NR	10, R/S/P, 41 y, EDSS 4.2, DD NR					

Data presented as mean (SD) unless otherwise noted

β: Beta regression coefficient, ↑: increased, *AAN* American Academy of Neurology, *CPET* cardiopulmonary exercise testing, *DD* disease duration, *EDSS* expanded disability status scale, *f* female, *IPAQ* International Physical Activity Questionnaire, *m* male, *MS* multiple sclerosis, *n.s.* no significant, *NR* not reported, *P* primary progressive MS, *PA* physical activity, *R* relapsing–remitting MS, *RM* repetition maximum, *r* correlation coefficient, *S* secondary progressive MS, *y* years

^a^Median (IQR)

^b^Range

## Results

The search strategy led to 7017 identified articles. After deduplication, the titles and abstracts of 3790 articles were screened for eligibility. The remaining 127 studies were assessed for full-text screen. After applying the selection criteria, 84 articles were excluded (see Fig. 1 for detailed exclusion reasons). A total of 43 studies were included. The PRISMA flow diagram is provided in Fig. 1.

### Characteristics of included studies and quality assessment

A detailed description of study characteristics from all included studies can be found in Table 3 (RCTs, CTs), Table 4 (cross-sectional studies), and Table 5 (cohort studies, randomized parallel group trials). Due to the relatively short duration of existing exercise studies compared to clinical phase III trials, ranging from 4 to 26 weeks (mean duration of 13.4 weeks), only short-term effects were assessed in the included studies.

In the following sections, the studies´ evidence level classification is summarized to conclude the strength of evidence (level of confidence) and to give evidence-based recommendations (Table 2A and B) on (i) the effect of structured exercise interventions on clinical measures of disease activity and progression, and on (ii) interactions of the physical activity and fitness level with clinical measures of disease activity and progression.

### Exercise

In total, 19 clinical exercise studies (five of class II evidence, ten of class III evidence, four of class IV evidence) investigated the effect of an exercise intervention on clinical measures of disease activity and progression. Regarding RoB assessment, the five class II evidence RCTs achieved a mean score of 4.1/6 for ‘low RoB’, 0.57/6 for ‘unclear RoB’ and 0.72/6 for ‘high RoB’, whereas the remaining eight class III evidence RCTs achieved a mean score of 2.38/6 for ‘low RoB’, 2.75/6 for ‘unclear RoB’ and 0.87/6 for ‘high RoB’. Of the included (R)CTs, six studies applied aerobic exercise [12, 31–35], three resistance exercises [11, 36, 37], five combined aerobic and resistance exercises [38–42], and one study applied a mind–body exercise [43]. Of the included longitudinal cohort and parallel group studies, one applied combined resistance and aerobic exercise [44], two combined resistance and balance exercises [45, 46], and one both climbing and mind–body exercise [47].

Two class II, six class III, and three class IV studies [33, 34, 36, 38, 40–46] report non-significant effects of exercise on EDSS, while two class III studies and one class IV study reported a decreased EDSS after exercise [35, 37, 47]. Two class II studies and one class III study reported exercise-induced improvements in the MSFC score after resistance exercise, combined endurance and resistance exercise, and interactive exergaming [11, 31, 41]. Considering MRI measures, changes in the percentage of brain volume were not observed in two class II studies [11, 12], whereas another class III study reported an increase in the left pallidum volume after exercise [32]. Although one of the class II studies reported an increase in cortical thickness [11], no change was observed in the other study [12]. Of note, none of the two studies reported an exercise-induced change in lesion load. The annualized relapse rate was investigated only in one class II study by Langeskov-Christensen et al. [12] who revealed a lower rate in the exercise group.

Recommendations: Based on results from two class II and six class III studies, there is moderate confidence (level B) that the EDSS score remains unchanged over the exercise intervention period. Moderate confidence (level B) from two class II studies indicates that exercise does not decrease disease activity indicated by the change in lesion load. Due to results from two class II and one class III study, there is moderate confidence (level B) that exercise improves the MSFC in pwMS. Moreover, there is low confidence (level C) from one class II study that exercise decreases the annualized relapse rate. While results from two class II studies show moderate confidence (level B) that exercise has no effect on brain volume in pwMS, there is very low confidence (level U) for the effect of exercise to increase cortical thickness.

Therefore, it is likely that exercise neither decreases nor stabilizes the EDSS and lesion load, whereas the MSFC score is likely to be improved. The brain volume is not likely to be changed by exercise, meaning that exercise neither increases nor maintains brain volume. Further, it is possible that exercise decreases the annualized relapse rate. Due to inconsistency, current data is insufficient to determine the effect of exercise on cortical thickness.

### Physical activity

In total, eight cross-sectional studies (two of class III evidence, six of class IV evidence) and four cohort studies with follow-up measurement time points (all of class IV evidence) investigated the effect of the physical activity level on clinical measures of disease activity and progression. Physical activity was assessed subjectively by a questionnaire (GLTEQ or IPAQ) in three studies [48–50], while the remaining studies used devices (i.e., Fitbit^®^, ActiGraph^®^, Axivity AX3^®^, Actiwatch^®^) to report daily or weekly step counts in most of the cases.

Two class III studies [51, 52] and eight class IV studies [48–50, 53–57] report significant negative associations between the physical activity and the EDSS. With respect to MRI outcomes, Kalron et al. (class IV) reported an increased hippocampal volume in active compared to inactive pwMS, whereas all other brain regions were not affected [48]. Positive associations between the level of physical activity and volume of whole brain gray and white matter as well as deep gray matter structures such as the thalamus, caudatus, putamen, and hippocampus were observed by Klaren et al. [23] (class IV).

Recommendations: Due to the low evidence classification of ten studies (two class III, eight class IV) reporting negative associations between the physical activity level and the EDSS, there is very low confidence (level U) that a high level of physical activity slows down disease progression quantified by EDSS. Again, due to the low evidence classification of two studies (class IV), there is very low confidence (level U) that a high level of physical activity increases or maintains (region-specific) brain volume in pwMS.

Therefore, current data is insufficient to determine the effect of physical activity on EDSS and (region-specific) brain volume in pwMS.

### Fitness

In total, 12 cross-sectional studies (three of class III evidence, nine of class IV evidence) investigated the effect of the fitness level on clinical measures of disease activity and progression. CRF was quantified as VO_2peak_ across all studies, except for two studies that quantified the VO_2max_ [58, 59]. Strength was assessed in four studies as maximal strength with isokinetics [60], dynamometer [61, 62], or the one-repetition maximum [63].

One class III study [60] and six class IV studies [21, 62–66] report a negative association between the EDSS and VO_2peak_ or strength measures, respectively, while the two class IV studies that assessed VO_2max_ do not report an association [58, 59]. Considering MRI measures, increased gray matter volume in midline cortical structures (class III) and deep gray matter structures (class IV) was found in pwMS with a higher CRF [22, 67]. Fritz et al. [61] revealed a positive association between a summed strength score and the corticospinal area. Only one class III study assessed lesion load volume and reported a negative association with the CRF of pwMS [67].

Recommendations: Due to the low evidence classification of seven studies (one class III, six class IV) reporting negative associations between the fitness level and EDSS, there is very low confidence (level U) that a high fitness level slows down disease progression assessed by EDSS. Due to the low number and low evidence classification of studies investigating the association between the fitness level and MRI-based outcomes, there is very low confidence (level U) that the (region-specific) brain volume and the lesion load are increased (brain volume), decreased (lesion load), or maintained by a high fitness level.

Therefore, current data are insufficient to determine the effect of a high fitness level on the EDSS, (region-specific) brain volume and lesion load in pwMS.

## Discussion

This review is the first that used a systematic approach to summarize and rate the evidence of existing studies assessing the effect of exercise interventions on established clinical measures of disease progression and activity in pwMS, as well as the relationship between these measures and the patient´s physical activity and fitness level. Despite the predominantly negative associations between the physical activity/fitness levels and clinical measures, the overall very low confidence in the evidence of existing studies does not confirm the promotion of those parameters to beneficially affect clinical measures. Results from exercise studies are more conclusive, pointing to improvements of the MSFC score, while the EDSS score, lesion load, and (region specific) brain volume are (likely) to remain unchanged. A major issue in this context, which hampers the significance of results, is the studies´ power. The majority of studies either did not report on the a priori specification of primary/secondary outcomes (10 (R)CTs, 8 NCTs, 19 crosssectional studies) or only analyzed the assessed clinical measures as secondary outcomes (4 RCTs). Only two (1 RCTs, 1 cross-sectional study) [12, 51] out of the 43 included studies specified them as a primary outcome. The fact that five RCTs did not include more than ten participants per group [37–40, 43] further substantiates the small power of existing RCTs. Of note, outcomes that are commonly used in phase III trials to determine disease activity (i.e., annualized relapse rate and changes in T2-hyperintense or gadolinium-enhancing T1 lesion load) [10] are rarely assessed in the reviewed studies.

Since the implementation and promotion of therapeutic interventions are based on the level of evidence, these findings support recently raised concerns about effective exercise promotion for pwMS due to the relatively low quality of studies in the field [5, 6]. The results of this systematic review underline the need to improve quality of RCTs and to rethink frequently used study designs to build new and increase existing evidence which is necessary to prove the proposed effects of exercise interventions and physical activity as (supportive) disease-modifying therapy options outlined in recent expert and narrative reviews [5, 6].

### Physical activity

The promotion of an active lifestyle is a major issue in the treatment of a broad range of diseases that are tightly linked to metabolic and immune-mediated disarrangement, including autoimmune diseases such as MS [19, 20]. With regard to MS, a recent article highlights the importance of physical activity and its promotion [24], since current evidence confirms that pwMS are still physically less active than the healthy population, although guidelines have been developed [17, 18]. Despite many limitations that are associated with the EDSS, i.e., a high intra- and inter-rater variability, non-linearity, and a limited responsiveness [10], the EDSS still represents the most frequently used clinical measure for disease progression and was assessed in ten out of the twelve included studies. All of the studies show a negative association between the physical activity level and the EDSS which means that pwMS with a higher EDSS are less physically active. However, this seems plausible in a way that neuromuscular functioning decreases with disease progression [68], leading to the need of a walking aid when the score is around six. Further, it should be considered that there is an increase in fatigue with a higher level of disability, which in turn may decrease the motivation to be physically active or to engage in exercise [69]. Positive associations have also been observed between the physical activity level and MRI-based outcomes. Physically more active pwMS showed increased volumes of gray matter including various subcortical brain regions of which the hippocampus region seems particularly sensitive [23, 48]. This is in line with evidence from preclinical animal models and human studies that support the beneficial effect of aerobic exercise on the hippocampus region [70, 71]. Although these findings are important and provide a clear rationale to determine effects of changes in physical activity on clinical measures of disease progression and activity longitudinally, it remains elusive why only a limited number of studies integrated follow-up measurements [49, 53, 57, 72]. A pilot study of Bahmani et al. [49] showed that vigorous physical activity, assessed by questionnaire, decreased during the first 2 years of disease, although not being associated with changes in the EDSS. Since this study covers a sensitive time frame of the disease which is proposed being a window of opportunity in MS exercise therapy [26], these findings are of high importance. Another longitudinal study tracked the physical activity of pwMS with a median EDSS score of 4.0 for 1 year [53]. Participants with a clinically meaningful increase in disability during this period showed a reduced daily step count and, more interestingly, those with a baseline daily step count below the cohort median had higher odds of clinically meaningful disability worsening within this year. These results may be indicative of an important role of physical activity to prevent disease progression.

Taken together, the findings from cross-sectional studies are interesting and provide a rationale to investigate the effect of structured physical activity on clinical measures of disease activity and progression in well-designed randomized controlled studies. This is highly important, since only relying on cross-sectional studies does not move research in this field forward.

### Fitness

An increase in physical activity or engagement in exercise programs results in higher fitness, usually quantified as CRF or strength. A recent study showed that CRF is positively associated with total brain volume and local gray matter volumes such as the right hippocampal gyrus in healthy adults [73]. Another study revealed a positive association between an increased CRF and a reduced brain atrophy in early-stage Alzheimer disease compared to healthy adults [74]. Therefore, an increase in CRF might be of high relevance for pwMS. Indeed, pwMS with higher levels of VO_2peak/max_ or higher muscle strength showed increased gray matter volume including various subcortical brain regions [22, 67]. Prakash et al. [67] further demonstrated a negative association between CRF and lesion load volume. Interestingly, a recent RCT reported that increased CRF in pwMS following 24 weeks of aerobic exercise was associated with an increase in the gray matter parenchymal fraction [12], thereby underscoring findings from cross-sectional studies that assume potential neuroprotective effects [22, 67]. Corresponding to findings from studies assessing physical activity, 9 out of the 12 included studies used the EDSS for correlation analyses with the fitness level. As already discussed in the *Physical activity* section, the consistent negative associations between the EDSS score and the fitness level observed in existing cross-sectional studies may be reasonable, since a moderate to high fitness level is the result of an active lifestyle which in turn has been shown to be associated with decreased disease severity. So again, despite these consistent associations, well-designed studies assessing both changes in fitness and disease progression/activity outcomes are necessary to provide evidence of higher quality.

### Exercise

In contrast to the results from cross-sectional studies, exercise studies predominantly showed non-significant effects on the EDSS, even when studies lasted 24 weeks or longer [40, 41, 43]. Studies that reported improvements suffer from methodological issues such as no randomization or matching of groups [35], no inclusion of a passive control group and focus on EDSS sub-domains [47], or a rather low sample size [37, 47]. However, low sample sizes are also observed in studies that report no changes in the EDSS [38, 40, 43]. Of note, only 8 of the included 13 RCTs tested 15 or more pwMS per group (the other 5 RCTs did not include more than 10 participants per group [37–40, 43]) which underlines the small power of existing RCTs. The relatively short intervention period, ranging from 5 to 26 weeks, may hamper the interpretation of significant results. Dalgas et al. recommend conducting clinical exercise studies lasting more than 1 year [6]. A recent study revealed that assessing short-term disability progression over 3–6 months to estimate treatment effects may overestimate the accumulation of permanent disability by 24–30% [75]. In this regard, most of the outcome measures assessed in the reviewed studies (i.e., EDSS, relapse rate, percentage brain volume change) may not be sensitive enough to quantify changes over a relatively short time period of up to 6 months. Therefore, longer intervention periods with follow-up measurements at regular intervals are reasonable to elucidate (long-term) exercise-induced effects on clinical measures in pwMS. This would enable researchers to quantify other important outcome parameters which may be affected by exercise, e.g., the time and magnitude of recovery after relapses in relapsing–remitting MS. Since RCTs usually aim to determine significant improvements in the intervention group compared to the control group, it should be considered that this is not always reasonable for measures such as the EDSS, brain volume or lesion load. Especially in the context of progressive disease, it would be a therapeutic success if these clinical parameters would remain stable over the exercise intervention period which optimally lasts for two or more years.

It is worth mentioning that only one study considered objectively assessed relapses to calculate the relapse rate which represents an important outcome in phase III trials [12]. According to a survey that collected data on the annualized relapse frequency in an American population, 44.1% of the participants reported less than one relapse in the preceeding 2 years, whereas 35.5% reported 1–2 and 20.2% more than two relapses, respectively [76]. A time frame of 6 months or shorter might therefore be insufficient to assess a meaningful exercise-induced effect on the (medically confirmed) relapse frequency. Compared to clinically evident relapses, T2-hyperintense and gadolinium-enhancing T1 lesion formation can occur subclinically, thereby representing markers that could be identified in shorter periods of time [10]. That makes the assessment of lesion formation potentially more suitable to detect disease activity in exercise studies that last 6 months or shorter. However, following 24 weeks of progressive resistance or aerobic exercise, no changes in lesion load were observed [11, 12]. These studies did also not reveal changes in brain volume, and the relative cortical thickness was increased after exercise in only one of the two studies [11]. Brain atrophy accumulates very slowly and when assessed in clinical trials to determine treatment efficiency of drugs, follow-up time points of brain atrophy quantification are usually several years [77, 78]. Although 6 months of aerobic exercise has been shown to increase gray and white matter volumes in an aged population [13], this time frame still might be too short to determine meaningful effects in pwMS. In this regard, confounding variables such as age and disease duration need to be considered when investigating short-term effects of exercise on brain atrophy measures in pwMS. No study observed an exercise-induced positive modulation of the hippocampal structures [12, 32], although evidence from preclinical animal models and human studies support a beneficial effect of aerobic exercise on the hippocampus region [70, 71]. Again, exercise studies lasting more than 1 year might reveal larger and clinically meaningful effect sizes. But rather than aiming to increase (region-specific) brain volume, maintaining the exisiting brain volume by counteracting neurodegeneration represents another meaningful outcome to consider. In this regard, Kjølhede et al. [11] revealed a trend in whole brain volume preservation following progressive resistance exercise for 24 weeks.

After 12 weeks of interactive step training and 24 weeks of progressive resistance exercise, respectively, the MSFC was improved [11, 31]. Only Romberg et al. [41] conducted a study which lasted longer (26 weeks), resulting in an improvement of the MSFC as well. When looking more into the different dimensions of the MSFC, both Romberg et al. [41] and Kjølhede et al. [11] observed improvements in the timed 25-foot walk test and the nine-hole peg test (9-HPT) in the exercise compared to the passive control group, whereas no changes were observed in the paced auditory serial addition test (PASAT). Hoang et al. [31] included the symbol digit modalities test (SDMT) and the 10-meter walk test instead of the PASAT and the 25-foot walk test. Despite the selection of different tools to assess the same clinical dimensions, the results are consistent with those of the aforementioned studies. Here, only the 9-HPT and 10-meter walk test improved in the exercise group compared to the control group, with results from the SDMT remaining unchanged. These results indicate that the cognitive dimension of the MSFC, assessed by the PASAT or SDMT, is less responsive to exercise over three to 6 months than the functioning of upper and lower extremities. This is in line with a recent meta-analysis that does not support the efficacy of exercise training on global or domain-specific cognitive performance in pwMS [79].

### An early exercise approach

Since almost all drug-based disease-modifying therapies have been shown to be effective primarily in the early relapsing–remitting disease course [3], theoretical considerations came up addressing a potential window of opportunity for exercise as a supportive disease-modifying treatment early in the disease course [26]. In this regard, it is important to note that the mean disease duration of MS populations from included studies is 9.27 years for (R)CTs, 10.52 years for cross-sectional studies, and 13.27 years for cohort studies. Only Bahmani et al. [49] included newly diagnosed pwMS to record changes in the physical activity behavior over 2 years. Therefore, it may be reasonable to focus more on the disease duration of participants rather than discussing solely intensity, frequency, and duration of exercise regimens. Documentation of the medication at study onset as well as changes of medication during the study period is important to control for disease-modifying effects that are not attributable to exercise. From the included studies, however, only five RCTs and one cohort study report on the participants´ medication status [11, 12, 31, 37, 38, 49].

### Potential exercise-induced mechanisms of action

From an evolutionary perspective, the human physiology is inherently associated with a moderate to high level of physical activity which affects many interconnected or rather soluble cellular systems such as central nervous system (CNS) structures and the immune system [19, 80]. Since MS is a neuroinflammatory disease, mitigation of both peripheral/central inflammation and neurodegeneration is of high importance. In this context, exercise represents a proposed therapy approach without side effects [5, 6]. Three weeks of exercise during an inpatient rehabilitation have been shown to decrease the systematic inflammatory index and the neutrophil-to-lymphocyte ratio in pwMS [81], the latter being associated with disease-specific symptoms, the EDSS, and disease activity [82, 83]. Interestingly, only high-intensity interval training reached significant results compared to moderate exercise. The same dose-dependent effect was shown for the reduction of matrix metalloproteinase-2 in another study with a similar study design [8], assuming that a higher cardiorespiratory stimulus over 3 weeks beneficially modulates blood–brain barrier integrity and decreases translocation of inflammatory immune cells into the CNS. Corresponding to these findings, results from a mouse model of experimental MS revealed exercise-induced inhibition of a decreased tight junction protein expression in the CNS observed in non-exercising mice [16]. Another animal study revealed significantly reduced demyelination and infiltration of proinflammatory Th17 cells into the CNS, whereas anti-inflammatory regulatory CD4^+^ T cells were enriched [15]. Again, high-intensity exercise was superior to moderate exercise, thereby adding evidence that a high cardiovascular stimulus over a certain time period may be superior to reach anti-inflammatory and neuroprotective effects. The exercise-induced intermittent metabolic stress is proposed to enhance neuronal survival, resilience, and plasticity through ketone body-mediated signaling [71]. The increased expression of the brain-derived neurotrophic factor plays an important role in this context. Indeed, increases in serum levels of brain-derived neurotrophic factor, also observed in pwMS in response to exercise [9], may be the result of epigenetic alterations induced by ketone bodies [84].

Another potential mechanism that may underlie exercise-mediated benefits in pwMS is the elevated metabolic flux of tryptophan degradation toward the immunosuppressive and neuroprotective end product kynurenic acid. Animal [85] and human [86] studies have demonstrated that exercise increases the flux along the metabolic kynurenine pathway to kynurenic acid, thereby preventing a pathological accumulation of kynurenine and quinolinic acid as well as increasing the availability of anti-inflammatory mediators. However, a better understanding of exercise-induced kynurenine pathway alterations in pwMS is needed to conclude on its contribution to improvements in symptoms or possibly also in disease progression.

Despite the increasing number of publications in the field, this review identified a lack of high quality evidence that exercise exerts consistent beneficial effects on several clinical measures of disease progression and activity. This could be due to the low sample size, heterogeneity of the included study population, and short duration of existing studies. Indeed, due to the short duration (averageing 13.4 weeks) compared to clinical phase III trials, only short-term effects were assessed. This raises the question if the available studies are appropriate to address the research question whether exercise affects established clinical measures of disease activity and progression in pwMS, since longer observations periods are needed to validly evaluate exercise-induced effects the relapse frequency, brain volume changes, or the EDSS score as discussed above. Further, only one exercise study assessed clinical measures of disease progression and activity as a primary outcome [12] which substantiates the underpowering of existing RCTs. In addition, the outcomes mainly do not correspond to outcomes used in phase III clinical trials to quantify disease progression or activity such as the relapse rate, changes in Gadolinium-enhancing T1- or T2-hyperintense lesion load, or brain atrophy [10]. Surprisingly, the MSFC was not considered in any cross-sectional or cohort study despite the fact that this clinical measure is commonly used in clinical trials and shows profound correlation with other clinically relevant measures such as the EDSS, relapse rate, white matter lesion load, and brain volumetric measures [10]. Despite the abundance of cross-sectional studies indicating beneficial effects of a high level of physical activity or fitness level on clinical measures of MS, it needs be considered that there is no causality within these associations and that negative associations between the EDSS and the physical activity or fitness level are somehow reasonable as discussed respectively in the “*Physical activity*” and “*Fitness*” sections within the discussion.

## Limitations

There are several limitations to this systematic review. First, we did not assess the risk of bias for included non-randomized controlled, non-controlled, and cross-sectional studies. Second, studies that examined the effect of a predominantly supportive exercise intervention or mainly consider task-oriented concise limb movements to improve activities of daily living were not included. Third, according to the criteria for rating therapeutic studies in the AAN Guideline Manual, it is not possible to rate a clinical exercise study to be of class I evidence, since a triple-masked study design is not applicable for randomized controlled exercise trials. This may hamper interpretation of results. Further, since the study duration does not represent a quality criterium for evidence classification in the standards published by the AAN, the relatively short duration of existing clinical exercise studies compared to phase III clinical trials needs to be considered. Fourth, the study population of included studies is not uniform regarding the disease subtype, meaning that most studies included pwMS of both the relapsing–remitting and a progressive form. Some studies did not report on the disease subtype of included participants. That makes it difficult to attribute exercise-induced effects on clinical measures of disease activity and progression to the different disease subtypes.

## Conclusion

The role of exercise interventions and physical activity to limit disease progression and activity is increasingly discussed. By systematically reviewing and rating the literature according to the AAN guidelines to draw evidence-based conclusions, this review indicates that exercise positively affects the MSFC score, while the EDSS score, lesion load, and (region-specific) brain volume are likely not affected. Despite consistent negative associations between the physical activity/fitness levels and clinical measures of disease progression and activity in pwMS, the overall very low evidence quality of existing studies makes it necessary to validate the results by conducting well-designed RCTs to advance research in this field. Methodological limitations such as missing a priori outcome specification, low sample size, and a predominantly “late timing” of exercise should be overcome in future studies. Since clinical phase III trials normally last 2 years or longer, the relatively short study duration of exisiting exercise studies represents a major limitation. Although well-designed clinical trials that last up to 12 months and assess important clinical measures are on the way (NCT03322761, NCT04762342), more RCTs assessing different primary end points of diseases activity or progression with long-term follow-up time points are needed to prove if engagement in regular exercise as well as increased physical activity is capable of exerting disease-modifying effects in pwMS.

## Supplementary Information

Below is the link to the electronic supplementary material.

Supplementary file1 (DOCX 96 KB)

## Data Availability

Data not provided in the manuscript are available either in the supplementary material or on request from the authors.
