# Supplementary material for: Fitness, physical activity, and exercise in multiple sclerosis: a systematic review on current evidence for interactions with disease activity and progression
Source: J Neurol. 2022 Jan 27;269(6):2922–40. doi: 10.1007/s00415-021-10935-6 (PMC9119898; doi:10.1007/s00415-021-10935-6)

**SUPPLEMENTARY MATERIAL**

Journal name:

Journal of Neurology

Article title:

Fitness, Physical activity, and Exercise in Multiple Sclerosis – A systematic review on current evidence for interactions with disease activity and progression.

Sebastian Proschinger^1†^, Puya Kuhwand^2†^, Annette Rademacher^1^, David Walzik^3^, Clemens Warnke^4^, Philipp Zimmer^3#^, Niklas Joisten^3#^

^1^Department for molecular and cellular sports medicine, Institute of cardiovascular research and sports medicine, German Sport University, Cologne, Germany

^2^Medical Faculty, University Hospital Cologne, Germany

^3^Marianne-Strauß-Klinik, Behandlungszentrum Kempfenhausen für Multiple Sklerose Kranke gGmbH, Berg, Germany

^4^TU Dortmund University, Institute for Sport and Sport Science, Division of Performance and Health (Sports Medicine), Dortmund, Germany

^5^Department of Neurology, University Hospital Cologne, Germany

^†^Shared first authorship

^#^Shared last authorship

Correspondence to

Prof. Philipp Zimmer

[philipp.zimmer@tu-dortmund.de](mailto:philipp.zimmer@tu-dortmund.de)

Phone: +49 231 755 7436

Fax: +49 231 755-4105

**Tab S1** Search String for the screened databases.

| Databases | Search string |
| --- | --- |
| MEDLINE, EMBASE, CINAHL, SPORTDiscus | (“Multiple sclerosis” [Title/Abstract] OR MS [Title/Abstract] OR “experimental autoimmune encephalomyelitis” [Title/Abstract] OR EAE [Title/Abstract]) AND (exercise [Title/Abstract] OR “physical training” [Title/Abstract] OR “physical activity” [Title/Abstract] OR fitness [Title/Abstract] OR “performance capacity” [Title/Abstract] OR sport [Title/Abstract] OR aerobic [Title/Abstract] OR running [Title/Abstract] OR swimming [Title/Abstract] OR walking [Title/Abstract] OR cycling [Title/Abstract] OR workout [Title/Abstract] OR ergometer [Title/Abstract] OR endurance [Title/Abstract] OR strength [Title/Abstract] OR resistance [Title/Abstract]) AND (MRI [Title/Abstract] OR lesion [Title/Abstract] OR “axonal loss” [Title/Abstract] OR brain volume [Title/Abstract] OR imaging [Title/Abstract] OR “magnetic resonance imaging” [Title/Abstract] OR “brain atrophy” [Title/Abstract] OR “disease progression” [Title/Abstract] OR “disease severity” [Title/Abstract] OR disability [Title/Abstract] OR relapse [Title/Abstract] OR “disease course” OR “clinical score” [Title/Abstract] OR EDSS [Title/Abstract] OR “neurological score” [Title/Abstract]) |

Initially, the scope of studies also comprised animal models of experimental autoimmune encephalomyelitis. After title and abstract screening, we decided to exclude these studies to increase homogeneity as well as clinical relevance of included studies. To not discard this animal-based research, another systematic review is currently in progress (PROSPERO registration number: CRD42021262092).

**Tab S2** Cochrane Risk of Bias rating for included randomized controlled trials


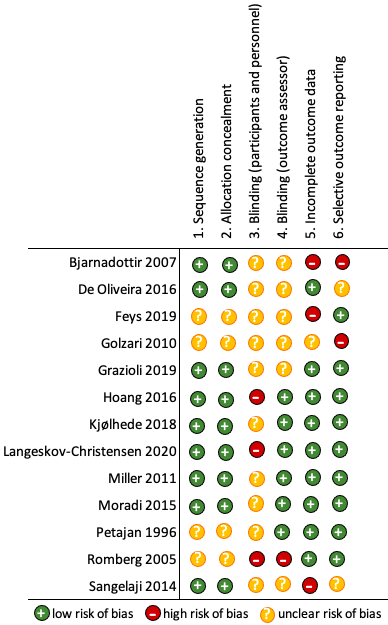

Supplement: Supplementary file 1 — Supplementary file1 (DOCX 96 KB) [file 415_2021_10935_MOESM1_ESM.docx]
